# Optimization of long-range PCR protocol to prepare filaggrin exon 3 libraries for PacBio long-read sequencing

**DOI:** 10.1007/s11033-022-08170-x

**Published:** 2023-01-24

**Authors:** Chiara Mareso, Elena Albion, William Cozza, Benedetta Tanzi, Stefano Cecchin, Paolo Gisondi, Sandro Michelini, Francesco Bellinato, Serena Michelini, Silvia Michelini, Matteo Bertelli, Giuseppe Marceddu

**Affiliations:** 1 Diagnostics Unit, MAGI EUREGIO, 39100 Bolzano, Italy; 2 Diagnostics Unit, MAGI’S LAB, 38068 Rovereto, Italy; 3 Section of Dermatology and Venereology, Department of Medicine, University of Verona, 37129 Verona, Italy; 4 Vascular Diagnostics and Rehabilitation Service, Marino Hospital, ASL Roma 6, 00047 Marino, Italy; 5 Unit of Physical Medicine, “Sapienza” University of Rome, 00189 Rome, Italy; 6 Unit of Neurosurgery, University of “Tor Vergata”, 00133 Rome, Italy

**Keywords:** Long-range PCR, *FLG*, PacBio, SMRTbell library, Protocol optimization, NGS

## Abstract

**Background:**

The filaggrin (FLG) protein, encoded by the *FLG* gene, is an intermediate filament-associated protein that plays a crucial role in the terminal stages of human epidermal differentiation. Loss-of-function mutations in the *FLG* exon 3 have been associated with skin diseases. The identification of causative mutations is challenging, due to the high sequence homology within its exon 3 (12,753 bp), which includes 10 to 12 filaggrin tandem repeats. With this study we aimed to obtain the whole FLG exon 3 sequence through PacBio technology, once 13-kb amplicons have been generated.

**Methods and results:**

For the preparation of SMRTbell libraries to be sequenced using PacBio technology, we focused on optimizing a 2-step long-range PCR protocol to generate 13-kb amplicons covering the whole *FLG* exon 3 sequence. The performance of three long-range DNA polymerases was assessed in an attempt to improve the PCR conditions required for the enzymes to function properly. We focused on optimization of the input template DNA concentration and thermocycling parameters to correctly amplify the entire *FLG* exon 3 sequence, minimizing non-specific amplification.

**Conclusions:**

Taken together, our findings suggested that the PrimeSTAR protocol is suitable for producing the amplicons of the 13-kb FLG whole exon 3 to prepare SMRTbell libraries. We suggest that sequencing the generated amplicons may be useful for identifying LoF variants that are causative of the patients’ disorders.

**Supplementary Information:**

The online version contains supplementary material available at 10.1007/s11033-022-08170-x.

## Introduction

**Fig. 1:**
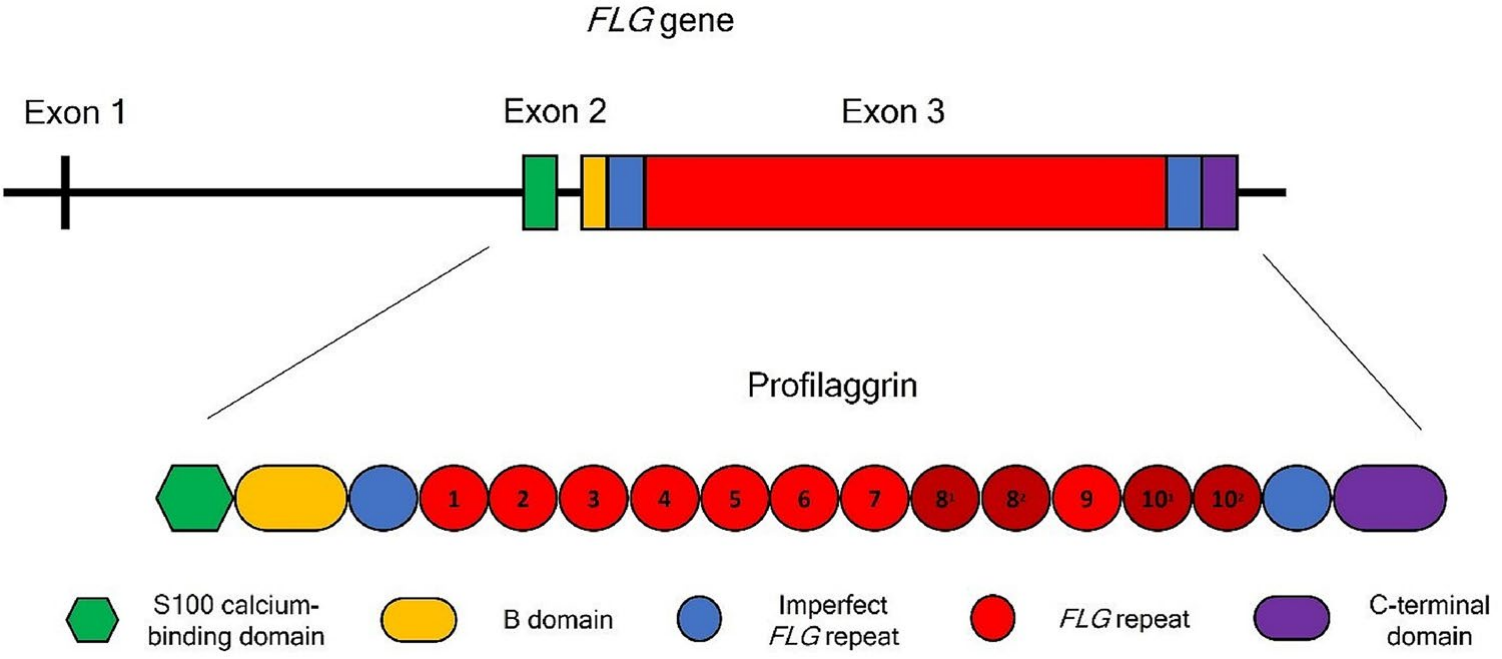
Scheme of FLG gene structure and profilaggrin organization, generated in PowerPoint Version 2021

The human filaggrin gene (*FLG*) is located on chromosome 1q21.3 in the epidermal differentiation complex (EDC) [1] and encodes profilaggrin, an insoluble highly phosphorylated ~ 400 kDa precursor polyprotein. When keratinocytes of granular layers turn into corneocytes, profilaggrin is dephosphorylated and processed by serine proteases, to yield multiple 37 kDa-filaggrin monomers [2]. Filaggrin is an intermediate filament-associated protein [3] that binds and organizes keratin intermediate filaments [4]. It promotes cornified-envelope formation to ensure appropriate barrier function [5], and once degraded it provides free amino acids [6], forming the natural moisturizing factor (NMF) [7], which helps maintaining epidermal hydration, and urocanic acid, which plays a role in protecting against UV radiation and in modulating immune functions [8, 9]. The human *FLG* gene has an unusually large exon 3, which involves a series of related but nonidentical 972 bp filaggrin tandem repeats, flanked by partial, imperfect filaggrin repeats [10]. The size of *FLG* gene exon 3 varies from 12.7 to 14.7 kb in the population, depending on repeat number, which ranges from 10 to 12 [11, 12]. It encodes most of the N-terminal domain and complete filaggrin monomers, each of 324 amino acids [1] (Fig. 1). Loss-of-function (LoF) mutations in this exon have been associated with two common inherited keratinization disorders, ichthyosis vulgaris (OMIM 146,700) and atopic dermatitis (ATOD2) (OMIM 605,803), and with a possible predisposition to food allergies, asthma and allergic rhinitis [13, 14].

Due to high sequence homology between tandem repeated regions of *FLG* exon 3 (see Supplemental Fig. S1), the identification of LoF variants in this exon is challenging, as short-sequencing reads map with low quality [15]. To date, most studies aimed at FLG genotyping have screened for common LoF mutations but have avoided sequencing the entire FLG coding region, which may result in underreporting the genetic contribution. The FLG exon 3 sequencing through NGS would result too complicated to be performed, due to the limits of NGS technology to analyze low complexity regions with high homology [10]. The NGS limitations in the analysis of genomic regions are not only limited to the FLG exon 3 sequence. As a matter of fact, it is well documented that NGS sequencing technology presents limitations in the mapping of different genomic regions, despite the use of sophisticated bioinformatics tools [16]. These regions include those which present base modifications in repetitive regions, as well as regions with structural variations, very high GC content and sequences with multiple homologous elements [17, 18].

To overcome limitations of “short-read sequencing” applications, Third-Generation sequencing (TGS), like PacBio methodologies, were developed through time, as they permit long-reads production with an average length of more than 10 kb. “Long-reads sequencing” is more representative of chromosomes, and consequently produce more contiguous genome reconstruction. They also permit to identify even the more complex variations and structural changes. In addition, the absence of PCR amplification in TGS approaches allows less biases and more homogeneous genome coverage. TGS library construction is simplified and less time-consuming if compared to the short-reads methods. [19–21]. In the FLG exon 3 specific case, the sequencing through PacBio technology enables highly accurate (99.8%) long high-fidelity (HiFi) reads [22] spanning the whole exon sequence, and can possibly identify even less common and less studied LoF variants.

In addition, TGS methods are shown to be more advantageous for long-reads in the sequence alignment phase, if compared to NGS methodologies. It is well known that, NGS technologies present strong limitations in sequencing long-reads of DNA sequences, due to the high number of biases introduced in the alignment phase. The sequenced strand of DNA pass through the fragmentation and amplification phases which lead to random clones that must be assembled into single continuous sequences, leading to high bias rates. In addition, NGS methodologies fail to generate a sufficient overlap between the DNA fragments. In contrast, TGS methodologies reduce the possibility of these biases: they reduce amplification bias, and generate DNA fragments which present reasonable lengths, that permit better overlapping for better sequence assembly [23].

With regard to the bioinformatic analysis of the sequencing data obtained, we referred to our internally developed bioinformatic pipeline “PacMAGI” [24]. The workflow of such bioinformatic pipeline presents a quality control performed by using FastQC and MultiQC, an alignment phase with reference genotype, through the use of Pbmm2, and the generation of mapping statistics through Picard and Qualimap tool. “PacMAGI” calling phase includes variants calling through Longshot, SVs calling through PBSVs. “PacMAGI” variants are finally annotated, filtered and interpreted using VarSome.

Long-range PCR (LR-PCR) is the most common approach to enriching long DNA fragments, and when combined with sequencing it can be used to screen for genetic variants [25]. We succeeded in finding suitable conditions for amplifying *FLG* exon 3 and for developing a LR-PCR protocol specifically designed for the preparation of SMRTbell libraries to be sequenced using PacBio technology.

## Results

SMRTbell library preparation starts with two PCR-rounds. The first-round PCR aims to amplify the chromosomal segment of interest (on chromosome 1q21.3) using target-specific primers tailed to Forward/Reverse universal sequences, which allow annealing of Forward/Reverse Barcoded Universal Primers (BUP) provided by PacBio during the second-round of PCR, resulting in amplicon barcoding.

### Optimization of first-round PCR protocol

Phusion Hot Start II High-Fidelity DNA Polymerase, KAPA HiFi HotStart DNA Polymerase, and PrimeSTAR GXL DNA Polymerase were selected on the basis of their features as candidate enzymes to amplify the ~ 13 kb DNA fragment of interest, which spans the whole *FLG* exon 3 sequence. As suggested by PacBio guidelines, we started performing our assays using Phusion Hot Start II DNA Polymerase, which is an extremely processive Pyrococcus-like polymerase, having an error rate of 4.4 × 10^–7^. This enzyme is able to amplify long amplicons, such as 20 kb λ DNA, and was therefore ideal for amplifying the ~ 13 kb DNA fragment of interest. Since Phusion works better at high denaturation and annealing temperatures, because of the high salt concentration in the reaction buffer, denaturation temperature was set at 98 °C for a short time (30 s), as recommended. Annealing temperature was set at 65 °C, as determined using the suggested online tool ‘Tm calculator’ provided by Thermofisher (www.thermofisher.com/tmcalculator). For the second attempt at first-round PCR optimization, we chose KAPA HiFi HotStart DNA Polymerase, which is also widely used in next-generation sequencing (NGS) library preparation. It belongs to the DNA polymerase B-family, and is engineered to improve yield, speed, sensitivity and DNA affinity without requiring accessory proteins or DNA binding domains. KAPA is a high-fidelity polymerase having an error rate of 1 error per 3.6 × 10^6^ nucleotides incorporated, and is capable of amplifying DNA targets up to 15 kb or even 20 kb in the case of less complex targets; it was therefore chosen to amplify the ~ 13 kb target region of interest. KAPA HiFi HotStart ReadyMix has a higher salt concentration than conventional PCR ready-mixes, which affects primer annealing. The annealing temperature was therefore set at 64 °C, 5 °C higher than primer melting temperature (59 °C). For the two-assay series performed with Phusion and KAPA, extension time was calculated considering 1 min/kb and set at 13 min and 20 s. In the third series of assays, PrimeSTAR GXL DNA Polymerase was selected to amplify *FLG* gene exon 3. A study comparing six DNA polymerase performances in LR-PCR found that PrimeSTAR GXL Polymerase was the best, by virtue of its ability to amplify large amplicons, even of different sizes and melting temperatures, under identical PCR conditions [26]. It is a high-fidelity polymerase capable of amplifying DNA fragments, even ≥ 30 kb in length. In the case of 10 to 30 kb amplicons, the manufacturer’s guidelines suggest a two-step PCR, which only consists of a denaturing (98 °C for 10 s) and an extension step (68 °C for 10 min), skipping the annealing phase. To produce sufficient amplicons for downstream applications, all the prepared reaction mixes were allowed 100 ng of template DNA. Reaction mixtures and thermocycling conditions adopted for the first-round PCR are listed in Table 1.

**Table 1:** (a) The optimized protocol of the first-round LR-PCR using three different DNA polymerases. The optimized PrimeSTAR* PCR protocol was the first-round PCR protocol chosen for the preparation of FLG exon 3 SMRTbell libraries. (b) The optimized protocol of the second-round LR-PCR using PrimeSTAR* PCR protocol

LR-PCR		
a	First-round	
Enzyme	Reaction mixture	PCR conditions
Phusion	100 ng template DNA, 1.25 μl 10 μM primers, 12.5 μl 2× Phusion HS II HF Master Mix, and distilled water to 25 μl	98 °C 30 s
		30 cycles
		98 °C 10 s
		65 °C 15 s
		72 °C 13 min
		and 20 s
		72 °C for 7 min
		Hold at 4 °C
KAPA	100 ng template DNA, 0.75 μl 10 μM primers, 12.5 μl 2 × KAPA HiFi HotStart ReadyMix, and distilled water to 25 μl	95°C 3 min
		30 cycles
		98°C 20 s
		64°C 15 s
		72°C 13 min
		and 20 s
		72°C 13 min and 20 s
		Hold at 4 °C
PrimeSTAR*	100 ng template DNA, 0.5 μl 10 μM primers, 5 μl 5 × PrimeSTAR GXL Buffer, 2 μl dNTP, 0.5 μl PrimeSTAR® GXL DNA polymerase, and distilled water to 25 μl	25 cycles
		98°C 10 s
		68°C 10 min
		Hold at 4 °C

b	Second-round	
Enzyme	Reaction mixture	PCR conditions
PrimeSTAR	3 μl first-round PCR DNA, 1 μl Barcoded Universal Primers, 5 μl 5 × PrimeSTAR GXL Buffer, 2 μl dNTP, 0.5 μl PrimeSTAR® GXL DNA polymerase, and distilled water to 25 μl	7 cycles
		98 °C 10 s
		68 °C 10 min
		Hold at 4 °C

**Fig. 2:**
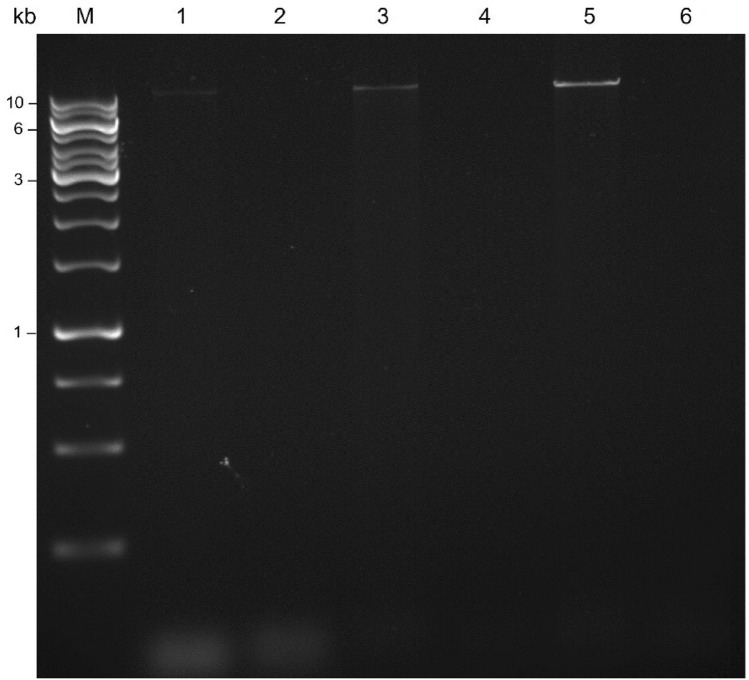
Gel electrophoresis of first-round PCR products, amplified using different polymerases. Lane M, DNA marker; 1, Phusion Hot Start II High-Fidelity DNA Polymerase; 3, KAPA HiFi HotStart DNA Polymerase; 5, PrimeSTAR GXL Polymerase; 2, 4, 6 were negative controls for the corresponding polymerases. The full-length gel is presented in Supplementary Fig. S2. The original unedited gel image is shown in the supplementary dataset, and the image was not joined from different parts of the gel

**Fig. 3:**
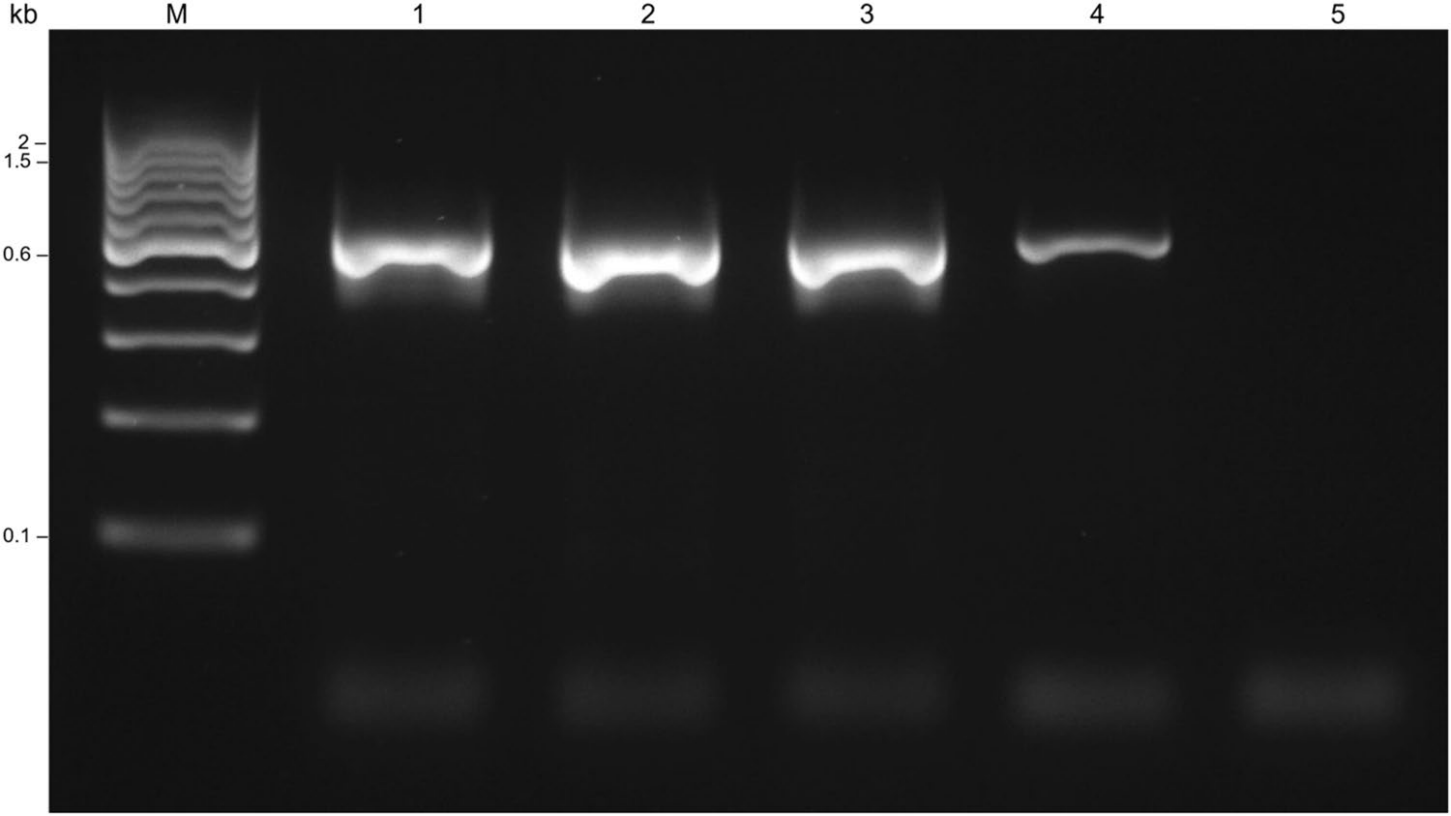
Gel electrophoresis of nested PCR products, performed on PCR products resulting from the previous tests and using different polymerases. Lane M, DNA marker; 1, Phusion Hot Start II High-Fidelity DNA Polymerase; 2, KAPA HiFi HotStart DNA Polymerase; 3, PrimeSTAR GXL Polymerase; 4, positive control; 5, negative control (5). The full-length gel is presented in Supplementary Fig. S3. Original unedited gel image is shown in the supplementary dataset and image was not joined from different parts of the gel

All the PCR products were loaded and run on 1 × agarose gel. The gel electrophoresis results (Fig. 2) showed that all three polymerases seemed to amplify *FLG* gene exon 3, since the corresponding bands on the agarose gel indicated amplification of a ~ 13 kb PCR product. To further confirm the results of the previous experiment, nested PCRs were performed starting from template DNA and on an aliquot of sample DNA, used as positive control. The resulting amplicons were run on 2% agarose gel, shown in Fig. 3. This test confirmed that the three previous assays effectively and correctly amplified *FLG* gene exon 3, since all gel bands proved amplification of the expected 473 bp in-target region of interest. Moreover, the intensity of the sample bands was higher than that of the gel band of the control DNA, indicating that the amplified sample target regions were at higher concentrations, since they had already been amplified in the previous LR-PCR. However, as shown in Fig. 2, the two ~ 13 kb gel bands of the PCR products amplified by Phusion and KAPA were very weak compared to that resulting from the amplification performed by PrimeSTAR, which produced amplicons at higher concentrations. We therefore chose the optimized PrimeSTAR PCR protocol (Table 1) to perform the second-round PCR for the preparation of *FLG* exon 3 SMRTbell libraries.

### Optimization of second-round PCR protocol

**Fig. 4:**
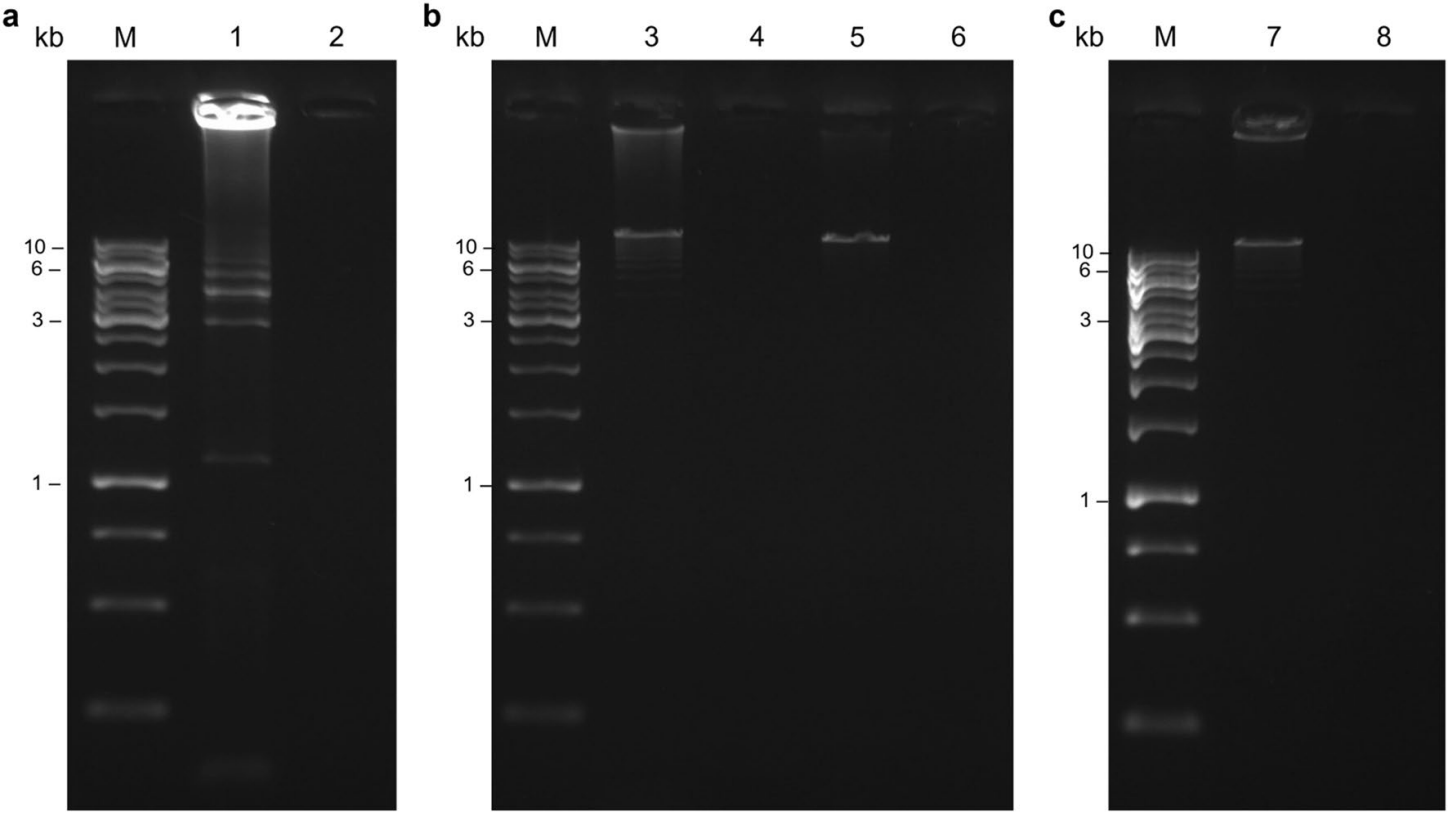
Gel electrophoresis of second-round PCR products amplified using PrimeSTAR GXL Polymerase, performed following the previously optimized LR-PCR protocol (**a**), and subsequently optimizing cycle number to 10 and 7 (**b**). Test perfomed using 3 μl of first-round PCR amplicons and 7 PCR cycles (**c**). Lane M, DNA marker; 1, 3, 5, 7 s-round PCR products; 2, 4, 6, 8 corresponding negative controls. The full-length gel is presented in Supplementary Fig. S4. Original unedited gel image is shown in the supplementary dataset and image was not joined from different parts of the gel

For the first attempt to optimize the second-round PCR, we used the same thermocycling conditions as for the previously optimized first-round PCR protocol. The gel electrophoresis results shown in Fig. 4a show a DNA smear pattern, indicating non-specific amplification, possibly due to the large number of cycles. Cycle number was therefore lowered from 25 to 10 (Figs. 4b lane 3), and from 10 to 7 (Figs. 4b lane 5) in a subsequent assay. The gel electrophoresis results (Fig. 4b) showed that the number of non-specific products decreased with decreasing cycle number. Due to the significantly reduced non-specific amplification resulting from the second assay, the cycle number was also kept at 7 in the subsequent test. The same test was performed starting with 3 μl of first-round PCR amplicons, to determine the appropriate amount of first-round PCR product to use and to increase final amplicon concentration. The resulting second-round PCR products were run on 1 × agarose gel. The gel image (Fig. 4c) showed very feeble gel bands related to nonspecific products close to the one of interest, which on the other hand was quite intense, indicating a high target amplicon concentration. The PCR product aliquot was purified with 0.45X AMPure PB beads, and DNA concentration was measured by Qubit dsDNA BR Assay Kit, performed with a Qubit Fluorometer. For SMRTbell library preparation 3000 ng of total input DNA is required, based on the amplicons size (~ 13 kb). Thus, samples were pooled in equimolar amounts, to obtain an equal representation of each amplicon in the data.

## Discussion

Several assays were performed to optimize the 2-step long-range PCR protocol for the preparation of *FLG* exon 3 SMRTbell libraries. Generating long amplicons in order to have the benefits of long-read PacBio sequencing for analyzing *FLG* exon 3 was challenging, due among other things to factors related to the target chromosomal region that includes GC content (55.3%) and highly homologous sequences. Suitable template DNA concentration and thermocycling conditions were determined according to amplicon length, primer melting temperature and reaction buffer composition. We assessed the performance of three long-range DNA polymerases, advertised as able to generate amplicons up to 20 kb or more. Compared to KAPA and Phusion, PrimeSTAR generated greater amounts of target amplicons, minimizing nonspecific product amplification. Unlike the other two polymerases, a two-step PCR protocol was adopted for PrimeSTAR. It did not include an annealing phase, but only denaturing (98 °C for 10 s) and extension (68 °C for 10 min) steps. The better performance of PrimeSTAR is probably explained by the fact that this enzyme was developed specifically to amplify products ≥ 30 kb in length, even if they are rich in GC-content [26]. The results showed that PrimeSTAR was suitable for producing *FLG* exon 3 amplicons for the preparation of SMRTbell libraries.

After a series of attempts to optimize the two-step LR-PCR protocol, we succeeded in amplifying the 13-kb genomic fragment spanning the entire sequence of *FLG* exon 3. This protocol was used to prepare SMRTbell libraries to sequence *FLG* exon 3 in 63 Italian patients with keratinization disorders. The quality of the results was determined, to verify the accuracy of protocol described above, which are reported in supplementary material [Supplementary FigS5; Supplementary material 2_MultiQC report for 63 patients].

Indeed, sequencing analysis of the entire *FLG* exon 3 at single nucleotide resolution would also enable screening for less common LoF variants, which may be more frequent in the Italian population. No treatment has yet been found for ichthyosis vulgaris and atopic dermatitis. There is only palliative care, like topical medications to ease symptoms including itch and pain, and to prevent these conditions from worsening. However, more comprehensive genetic testing using new generation technologies could offer patients the chance to be candidates for future therapeutic options.

## Methods

The SMRTbell library to sequence *FLG* gene exon 3 was prepared following the PacBio protocol guidelines Procedure & Checklist—Preparing SMRTbell® Libraries using PacBio® Barcoded Universal Primers for Multiplexing Amplicons—Part Number 101–791-800 Version 02 (April 2020) (https://www.pacb.com/wp-content/uploads/Procedure-Checklist-Preparing-SMRTbell-Libraries-using-PacBio-Barcoded-Universal-Primers-for-Multiplexing-Amplicons.pdf). All assays were performed using the same genomic DNA sample, derived from transformed reference cell lines provided by Coriell Institute for Medical Research as template. For the purpose of this work, we purchased the NA20509 cell line (INTERNATIONAL HAPMAP PROJECT—TOSCANI IN ITALIA (TUSCANS IN ITALY)) from Coriell Cell Repositories (http://ccr.coriell.org/). Reactions were performed using a SureCycler 8800 Thermal Cycler (Agilent). The first step of the SMRTbell library preparation workflow consists of two PCR-rounds, which require optimization according to the target region features.

For the first-round PCR, forward target-specific primer (5’-AGGGTTATTTTGAGCTCTTTGTGAA-3’) and reverse target-specific primer (5’-TGGGACAGTGATTATGTTGGAGA-3’) tailed to F/R universal sequences were designed to amplify the ~ 13 kb DNA fragment spanning the entire *FLG* exon 3 sequence. Primer design was performed using the freely available program Primer3web (https://primer3.ut.ee) and, to avoid mispriming, the primer couple was tested in silico using NCBI Primer-BLAST (https://www.ncbi.nlm.nih.gov/tools/primer-blast) and the UCSC tool In-Silico PCR (https://genome.ucsc.edu/cgi-bin/hgPcr). Three long-range DNA polymerases were selected to perform the LR-PCR, including Phusion Hot Start II DNA Polymerase (Thermo Scientific, Vilnius, Lithuania), KAPA HiFi HotStart DNA Polymerase (KAPA Biosystems, Cape Town, South Africa), and PrimeSTAR GXL DNA Polymerase (TaKaRa Bio, Shiga, Japan). For each selected polymerase, assays were optimized according to the manufacturers' guidelines and set up as shown in Table 1. To assess the results of LR-PCR amplification, PCR products were run on 1% agarose gel and visualized by staining with GelRed Nucleic Acid Gel Stain (Biotium, Hayward, CA). Image scanning was performed with GeneSys software version 1.5.4 (https://www.syngene.com/software/genesys-rapid-gel-image-capture/). To confirm that the amplicons were derived from *FLG* exon 3, nested PCRs were performed using in-target forward primer (5’-AGTGATAGTGAGGGACATTCAGAG-3’) and in-target reverse primer (5’-CCGTCTCCTGATTGTTTGTCCT-3’) to amplify a 473 bp DNA fragment inside the previously amplified target region. These reactions containing 1 μl of first-round PCR products, 1 μl of the two F/R primers (10 μM), 10 μl AmpliTaq Gold FastPCR Master Mix (Thermo Scientific, Vilnius, Lithuania) and distilled water up to 20 μl were heated to 95 °C for 10 min, followed by 30 cycles at 96 °C for 3 s, 59 °C for 3 s, and 68 °C for 10 s, and a final extension at 72 °C for 10 s.

The first attempt to optimize the second-round PCR protocol was performed mixing 1 μl of first-round PCR product, 1 μl BUP, 5 μl 5 × PrimeSTAR GXL Buffer, 2 μl dNTP, 0.5 μl PrimeSTAR® GXL DNA polymerase and distilled water up to 25 μl, using the same cycling conditions as for the previously optimized first-round PCR protocol (Table 1). Subsequently, second-round PCR protocol was optimized by decreasing cycle number to seven and increasing the amount of template DNA to 3 μl. Second-round PCR products were purified using 0.45X AMPure PB beads (Pacific Biosciences) and DNA concentrations were read on a Qubit 2.0 Fluorometer using the Qubit dsDNA Broad Range Assay Kit (Invitrogen Life Technologies, USA).

## Supplementary Information

Below is the link to the electronic supplementary material.

Supplementary file1 (HTML 1709 kb)

Supplementary file2 (DOCX 2560 kb)

## Data Availability

The dataset that was analysed during the current study is available in the Coriell Cell repository [https://catalog.coriell.org/0/Sections/Search/Sample_Detail.aspx?Ref=NA20509&Product=DNA].
