# Supplementary material for: Optimization of long-range PCR protocol to prepare filaggrin exon 3 libraries for PacBio long-read sequencing
Source: Mol Biol Rep. 2023 Jan 24;50(4):3119–27. doi: 10.1007/s11033-022-08170-x (PMC10042914; doi:10.1007/s11033-022-08170-x)
Supplement: Supplementary file 2 — Supplementary file2 (DOCX 2560 kb) [file 11033_2022_8170_MOESM2_ESM.docx]

**Supplementary FigS1.**

*Imp*

*Seq1*

*Seq2*

*Seq3*

*Seq4 Seq5*

*Seq6*

*Seq7*


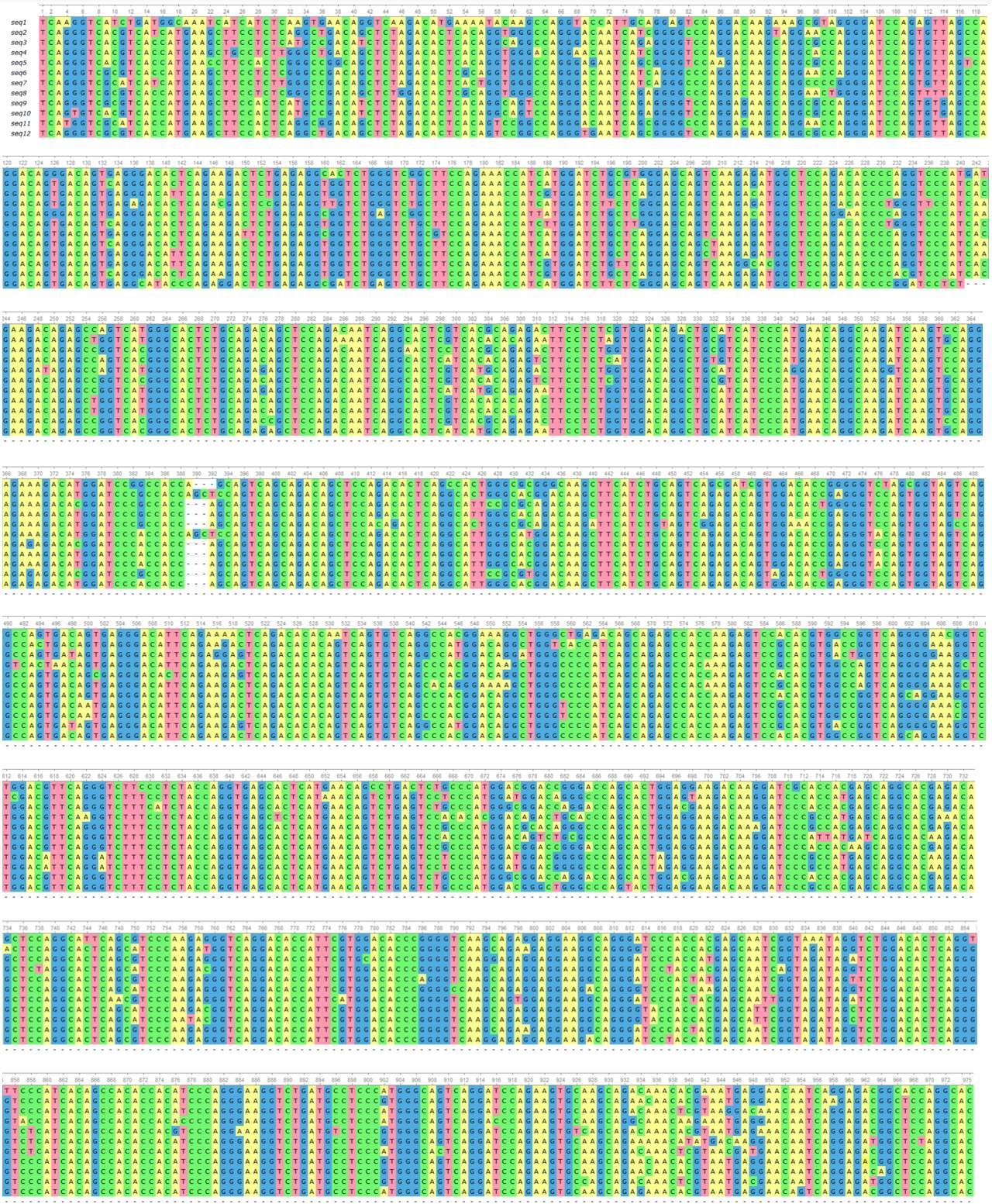


*Seq8*

*Seq9*

*Seq10*

*Imp*

**Supplementary FigS1** Sequence alignment of tandem-repeated homologous regions (*Seq1-10*), flanked by imperfect repeats (*Imp*) harboured in FLG gene exon 3, performed using Unipro UGENE software v36.0 64 -bit version http://ugene.net/

**Original images of full-length gels used in the main article, which were cropped**

No figures were cropped and joined together from different images. Contrast adjustment was applied equally across the entire images.


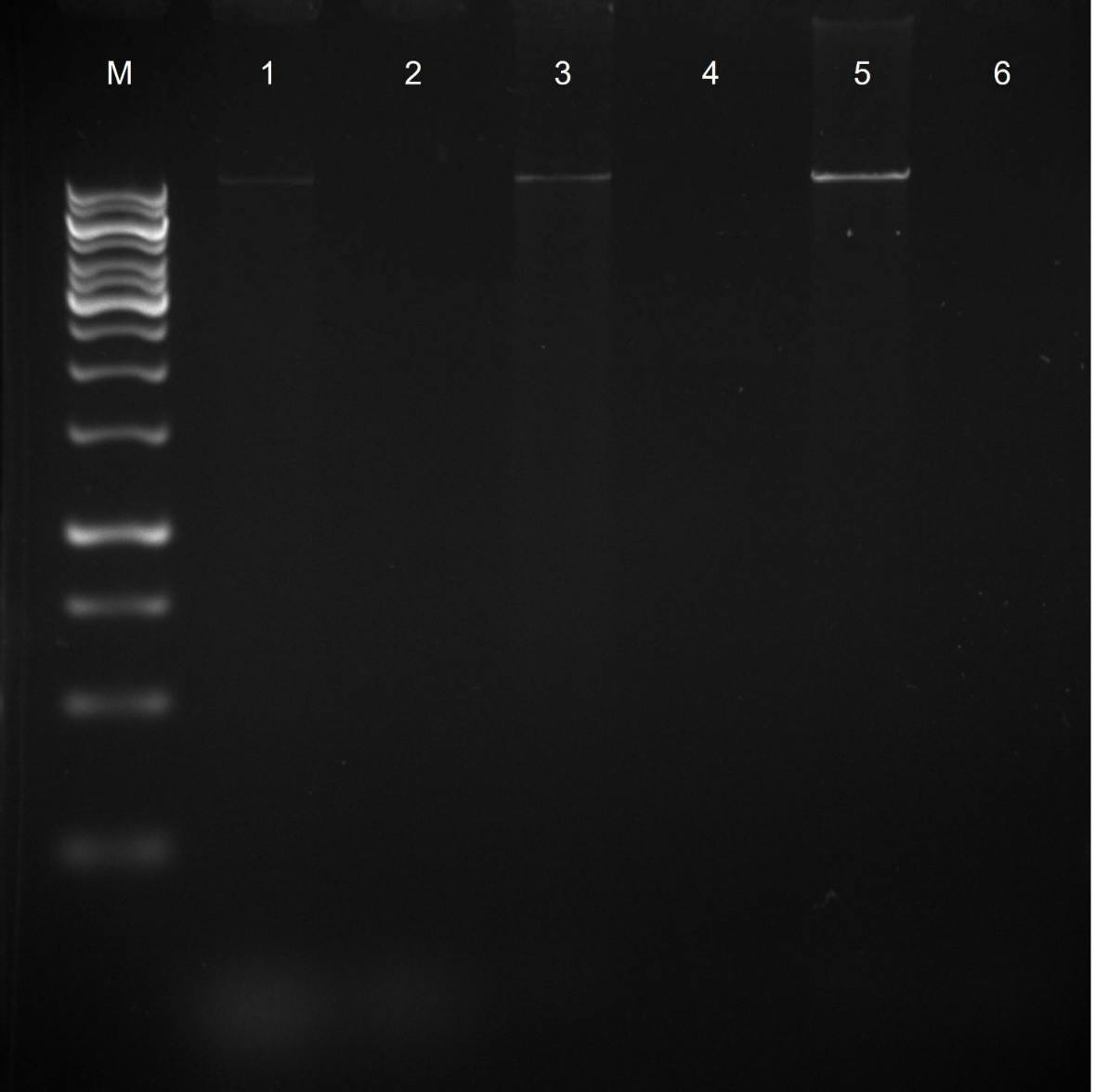


**Supplemetary FigS2** The full-length gel of Fig. 2 in the main article. Gel electrophoresis of first-round PCR products were amplified by using different polymerases. Lane M, DNA marker; 1, Phusion Hot Start II High-Fidelity DNA Polymerase; 3, KAPA HiFi HotStart DNA Polymerase; 5, PrimeSTAR GXL Polymerase; 2, 4, 6 were negative controls for the corresponding polymerases.


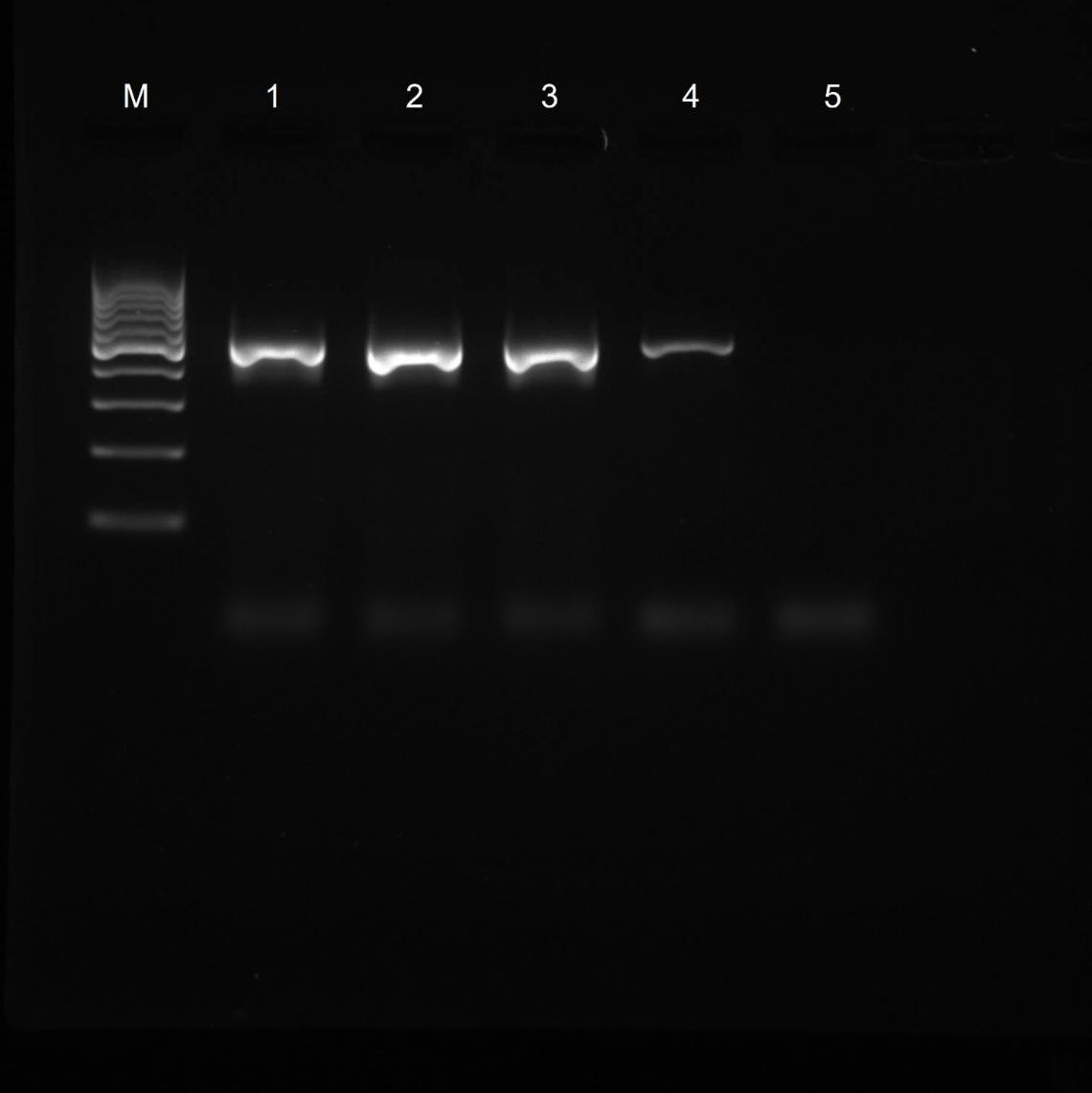


**Supplemetary FigS3** The full-length gel of Fig. 3 in the main article. Gel electrophoresis of nested PCR products were performed on PCR products resulting from the previous tests, using different polymerases. Lane M, DNA marker; 1, Phusion Hot Start II High-Fidelity DNA Polymerase; 2, KAPA HiFi HotStart DNA Polymerase; 3, PrimeSTAR GXL Polymerase; 4, positive control; 5, negative control (5).


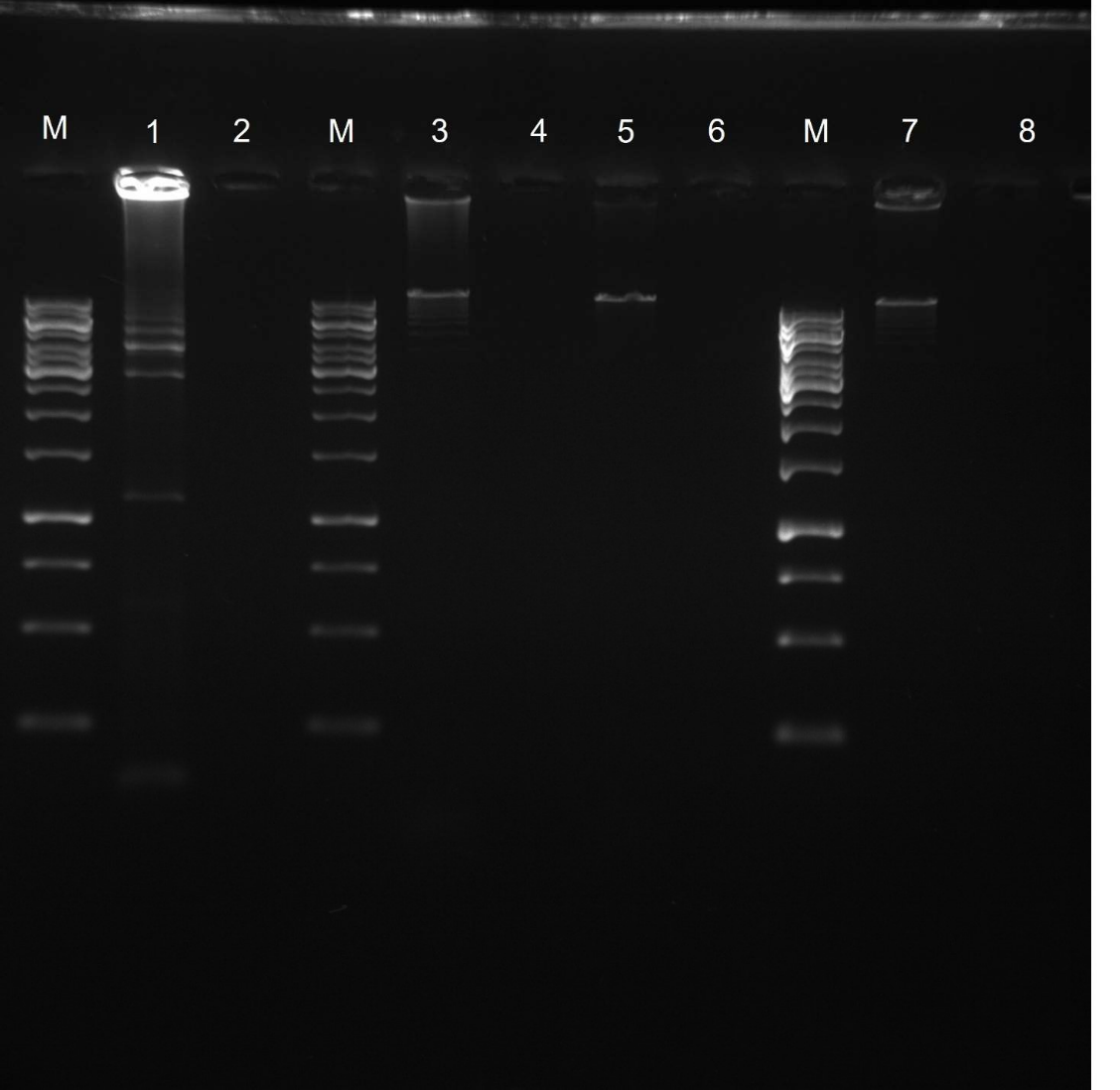


**Supplemetary FigS4** The full-length gel of Fig. 4 in the main article. Gel electrophoresis of second-round PCR products were amplified by using PrimeSTAR GXL Polymerase, performed following the previously optimized LR-PCR protocol (a), and subsequently optimizing cycle number to 10 and 7 (b) and initial DNA template amount (c). Lane M, DNA marker; 1, 3, 5, 7 second-round PCR products; 2, 4, 6, 8 corresponding negative controls.

**Supplemetary FigS5**


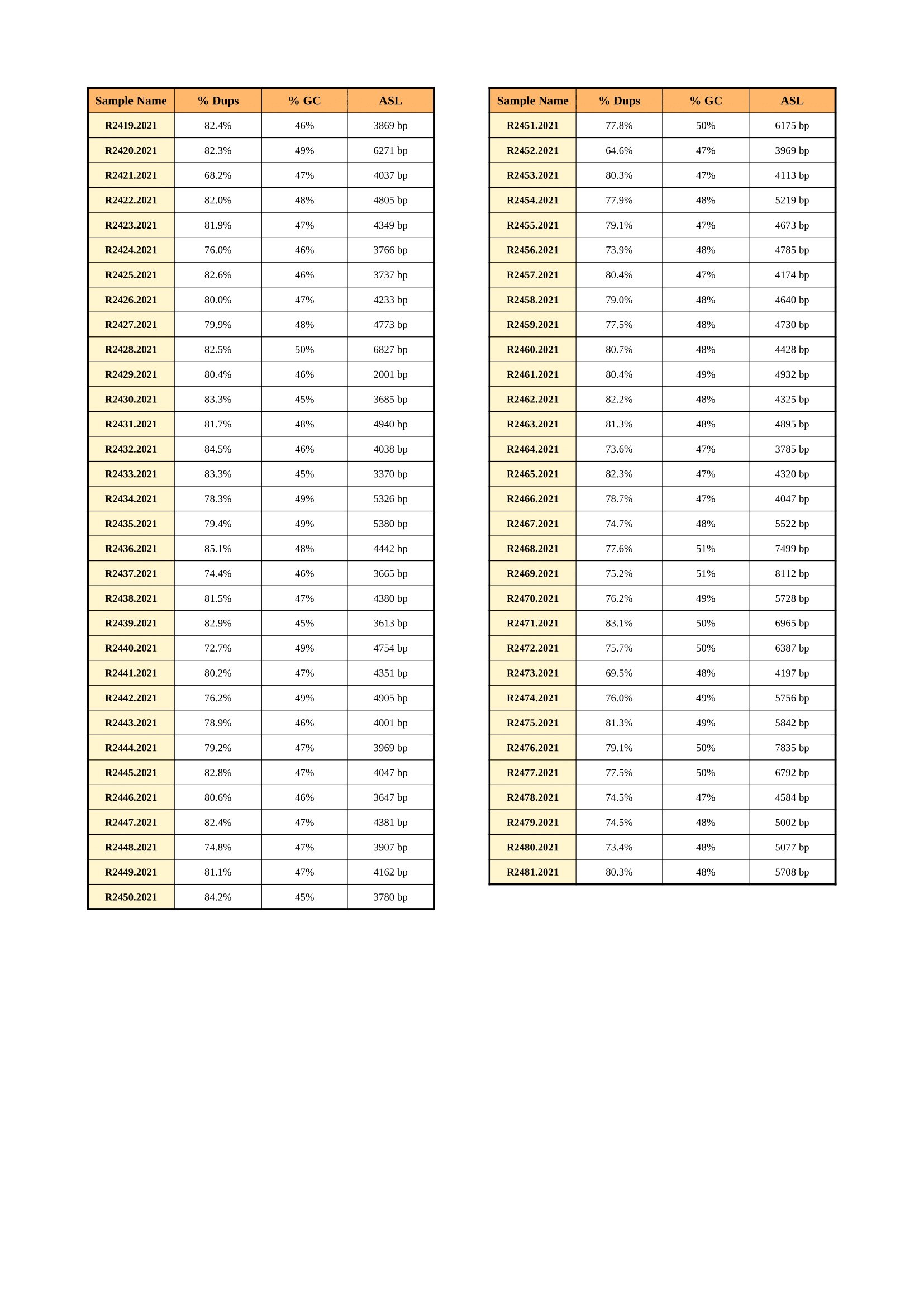


**Supplementary FigS5** The first column reports sample names registered as research IDs for the 63 patients analyzed. The second, the third and the fourth columns present the data calculated for duplicate reads, GC content and bp average sequence length, respectively. For further information we refer to Supplementary material 2_MultiQC report for 63 patients.
